# Parametric frailty models for clustered data with arbitrary censoring: application to effect of male circumcision on HPV clearance

**DOI:** 10.1186/1471-2288-10-40

**Published:** 2010-05-06

**Authors:** Xiangrong Kong, Kellie J Archer, Lawrence H Moulton, Ronald H Gray, Mei-Cheng Wang

**Affiliations:** 1 Department of Population, Family and Reproductive Health, Bloomberg School of Public Health, Johns Hopkins University, Baltimore, MD, USA; 2 Department of Biostatistics, School of Medicine, Virginia Commonwealth University, Richmond, VA, USA; 3 Department of Biostatistics, Bloomberg School of Public Health, Johns Hopkins University, Baltimore, MD, USA; 4 Department of International Health, Bloomberg School of Public Health, Johns Hopkins University, Baltimore, MD, USA

## Abstract

**Background:**

In epidemiological studies, subjects are often followed for a period during which study outcomes are measured at selected time points, such as by diagnostic testing performed on biological samples collected at each visit. Although test results may indicate the presence or absence of a disease or condition, they cannot provide information on when exactly it occurred. Such study designs generate arbitrarily censored time-to-event data, which can include left, interval and right censoring. Adding to this complexity, the data may be clustered such that observations within the same cluster are not independent, such as time to recovery of an infectious disease of family or community members. This data structure is observed when evaluating circumcision's effect on clearance of penile high risk human papillomavirus (HR-HPV) infections using data collected from the male circumcision(MC) trial conducted in Rakai, Uganda, where the multiple infections within individual and HPV testings performed at trial follow-up visits gave rise to the clustered data with arbitrary censoring.

**Methods:**

We describe the use of parametric proportional hazards frailty models and accelerated failure time frailty models to examine the relationship between explanatory variables and the survival outcomes that are subject to arbitrary censoring, while accounting for the correlation within clusters. Standard software such as SAS can be used for parameter estimation.

**Results:**

Circumcision's effect on HPV infection was a secondary end point in the Rakai MC trial, and HPV genotyping was conducted for penile samples of a subset of trial participants collected at enrollment, 6, 12 and 24-month follow up visits. At enrollment, 36.7% intervention arm men (immediate circumcision) and 36.6% control arm men (delayed circumcision at 2 years) were infected with HR-HPV, with the number of infections per man being 1-5. The proposed models were used to examine whether MC facilitated clearance of the prevalent infections. Results show that clearance of multiple infections within each man is highly correlated, and clearance was 60% faster if a man was circumcised.

**Conclusions:**

Parametric frailty models provide viable ways to study the relationship between exposure variables and clustered survival outcome that is subject to arbitrary censoring, as is often observed in HPV epidemiology studies.

## Background

In epidemiological studies, subjects are often followed over time and study outcomes are measured at selected time points. The study outcomes may be diagnostic testing results based on biological samples such as tissue, blood, or urine samples. The testing at each time point can detect the presence or absence of a condition (for example, infection of an infectious disease), but it does not provide the exact information of when the infection or condition occurred. The best knowledge about the actual event time is that it occurred during the interval where discordant test results are observed at the start and end of the interval, yielding so-called *interval *censoring of time-to-event (survival) data [1]. If the event is observed at the first follow-up, we only know that the event occurred before the first scheduled testing time, and this generates *left *censored data. On the other hand, if a subject drops out of the study or remains event free at the end of the study, the time to event could only be after the last observed testing time, which corresponds to the well-known *right *censoring. When it is of interest to evaluate the effect of treatment on time to event occurrence, many analysts use either the end point [2] or the mid-point of the interval where discordant results were observed as the actual event times. The former way introduces "time aggregation" bias when estimating the hazard rate, while the latter mid-point method reduces time-aggregation bias under certain conditions [3]. For unbiased estimation of the treatment or exposure effect, approaches that directly model the likelihood of the arbitrarily censored data (including left, interval and right censoring) can be used [4]. Other than the presence of arbitrarily censored data, what can further complicate the analysis is the presence of clustered data where the study subjects are correlated within each cluster, such as patients visiting the same clinic in a multi-site study, members within the same family or community, or repeated testing on the same subject. The Cox regression model has been extended for clustered time-to-event data to model the marginal distributions without full specification of the correlation structure between the clustered observations [5,6], though it has only been established for data with non-informative right censoring. For clustered data with interval censoring, [7] introduces the use of conditional proportional hazards model (i.e. semiparametric frailty model), and briefly discusses the advantages of parametric frailty models. In this paper we describe the use of parametric frailty models to assess treatment or exposure effect on time to a well-defined event for clustered survival data with arbitrary censoring, which includes left, right, as well as interval censoring. The model estimation can be carried out using existing software, such as SAS (SAS Institute, Inc., Cary, NC) PROC NLMIXED. The method is illustrated through the application on data collected from a randomized clinical trial of male circumcision (MC) conducted in Rakai, Uganda, and the current study purpose is to evaluate the effect of MC on clearance of penile high risk human papillomavirus (HR-HPV) in HIV-negative men.

The following section provides a brief introduction about the HR-HPV data to exemplify the problem of interest. Statistical notations and the proposed model are then presented through the context of the example. Parameter estimation can be realized using SAS and the SAS code for this example is provided. The analytical result on the HR-HPV data is subsequently presented. We conclude with discussion about the general use of the proposed model for clustered survival data with arbitrary censoring.

### Example: Effect of Male Circumcision on HR-HPV clearance

A clinical trial of MC was conducted on initially HIV-negative uncircumcised men aged 15-49 years in Rakai District of Uganda during 2003-2006 [8,9]. Approximately 5000 men were enrolled in the trial and randomized to either immediate circumcision (intervention arm) or delayed circumcision after 24 months (control arm). At enrollment and follow-ups scheduled at 6, 12, and 24 months, variables about participant sociodemographic characteristics, sexual risk behaviors and symptoms of sexually transmitted infections were recorded using questionnaires. Penile swabs were collected by clinicians from the preputial cavity of uncircumcised men and from the coronal sulcus/glans of circumcised men. Circumcision effect on HPV was a secondary endpoint in the trial and HPV testing was performed restricted to consistently HIV-negative married men with concurrently enrollment wives. HPV testing was performed on samples collected at all four visits only for a subset of randomly selected 330 such men (39.5%) in the intervention arm and 314 men (39.1%) in the control arm due to resource limitation. Roche HPV Linear Array (Roche Diagnostics, Indianapolis, IN) was used for HPV genotyping. The fourteen genotypes 16, 18, 31, 33, 35, 39, 45, 51, 52, 56, 58, 59, 66, and 68 were considered HR-HPV, that is, carcinogenic viral genotypes. Therefore, for each participant at each visit, there are fourteen binary indicators indicating the presence or absence of the fourteen HR-HPV genotypes, respectively. At enrollment, the intervention and control arm were comparable in sociodemographic and sexual behavioral characteristics [10]; the prevalence of HR-HPV was also comparable, with 36.7% men being positive on at least one HR-HPV genotype in the intervention arm and 36.6% in the control arm. One particular interest with this dataset is to study whether MC facilitates HR-HPV clearance process since foreskin provides a reservoire for viral protein expression. We studied circumcision's effect on clearance of enrollment prevalent HR-HPV infections, and the prevalent infection profile is summarized in Table 1 (upper panel).

**Table 1:** Enrollment prevalence of HR-HPV infections and observed clearance proportions in the intervention arm (I) and the control arm (C).

	Intervention n = 330 (%)	Control n = 314 (%)	Prevalence Rate Ratio (I/C)	95% Confidence Interval
No. of men infected with Any HR-HPV	121 (36.7)	115 (36.6)	1.00	(0.82-1.23)
Single HR-HPV	77 (23.3)	80 (25.5)	0.92	(0.70-1.20)
Multiple HR-HPVs	44 (13.3)	35 (11.1)	1.20	(0.79-1.81)

Total No. of infections No. of infections cleared	180	169		
by 6-month	112 (62.2)	98 (58.0)		
by 12-month	141 (78.3)	123 (72.8)		
by 24-month	171 (95.0)	161 (95.3)		

Clustered data structure arises in this dataset, as each participant (a cluster) had testing results for the fourteen HR-HPV genotypes. A person with multiple infections may contribute multiple events (i.e. clearances) that are likely to be highly correlated. The exact clearance time point is unavailable, but it is known to be either before the first subsequent visit (left censored), or lie within an interval between two visits (interval censored), or after the last follow-up visit (right-censored).

## Methods

### Notations

Let *T *denote the random variable for the time to event. Assume there are *n *clusters in the study, and the *i*th cluster (*i *= 1, 2, ⋯, *n*) has *j *= 1, 2, ⋯, *n*_*i *_observations. In the HR-HPV example, *T *is the time to clearance of an HR-HPV infection, and each participant is a cluster. For a person with single genotype HR-HPV infection, *n*_*i *_= 1; while if a person has multiple HR-HPV genotype infections, *n*_*i *_> 1, and the clearance of these multiple infections is not independent. For the *j*th HR-HPV genotype infection (*j *= 1, 2, ⋯, *n*_*i*_) on the *i*th participant, *t*_*ij *_is the actual clearance time which is not exactly observed. Let (*a*_*ij*_, *b*_*ij*_] denote the interval where the clearance occurs, i.e. *a*_*ij *_<*t*_*ij *_≤ *b*_*ij*_. If a prevalent infection of genotype *j *for person *i *is observed to be cleared at first follow-up, then *a*_*ij *_= 0, and *t*_*ij *_≤ *b*_*ij *_= 6 months, which corresponds to a left censored observation; if it is never cleared during the trial period, then *a*_*ij *_= 24 months (the last follow-up time), and *a*_*ij *_<*t*_*ij *_< ∞ (the upper bound *b*_*ij *_can be viewed as ∞), which is a right censored observation.

Suppose there are *p *explanatory variables, which can include the variable indicating treatment arm and other covariates that may be of interest, and let **x**_*ij *_denote the vector of explanatory variables for the *j*th infection in person *i*. Without loss of generality, we only consider one explanatory variable for treatment arm, and *x*_*ij *_= 1 denotes intervention and *x*_*ij *_= 0 denotes control. Note that treatment arm for the fourteen HR-HPV genotypes is identical for each participant, although in general applications, **x**_*ij *_can be different for different observations belonging to the same cluster.

### Parametric Proportional Hazards Frailty Model

Let *h*(*t*) denote the hazard function representing the "hazard" (i.e. the instantaneous rate) of clearance at time *t*. To examine the treatment effect on clearance, we can model the hazard to be a function of the explanatory variables, while at the same time including a random effect to account for the correlation between the multiple HR-HPV genotype infections on such infected participants. For example, as in [7], for clearance of the *j*th infection on person *i*, we can use the conditional proportional hazards model (or semiparametric frailty model):(1)

Without the ξ_*i *_term, expression 1 is just the ordinary Cox proportional hazards model, where *h*_*ij*_(*t;***x**) is the "hazard" for clearance of the *j*th genotype infection in person *i*, *h*_0_(*t*) is the baseline hazard for a person with all explanatory variables being zero. The *ξ*_*i *_term in expression 1 represents a random effect realized on the *i*th person. It is assumed to follow a prior distribution, such as a normal distribution, or equivalently exp(*ξ*_*i*_) ~ a log-normal distribution. The use of the normal random effect *ξ*_*i *_on *t *(through the hazard function) is one way of introducing correlation within the *i*th cluster, and is similar to that in linear mixed effects or generalized linear mixed effects models. Because the same realization value of *ξ*_*i *_(from its prior distribution) is shared by observations on the multiple HR-HPV genotype infections within the *i*th person (thus its' subscript does not depend on *j *which indexes genotype), it is therefore taken into account of the dependence between the clearance times of these multiple HR-HPV infections. One advantage of using normally distributed random effect is that more complicated correlation structures between observations, such as multi-level correlations, can be handled naturally by extending the use of a univariate normal random effect to multivariate normal random effects [11,12].

**Figure 1:**
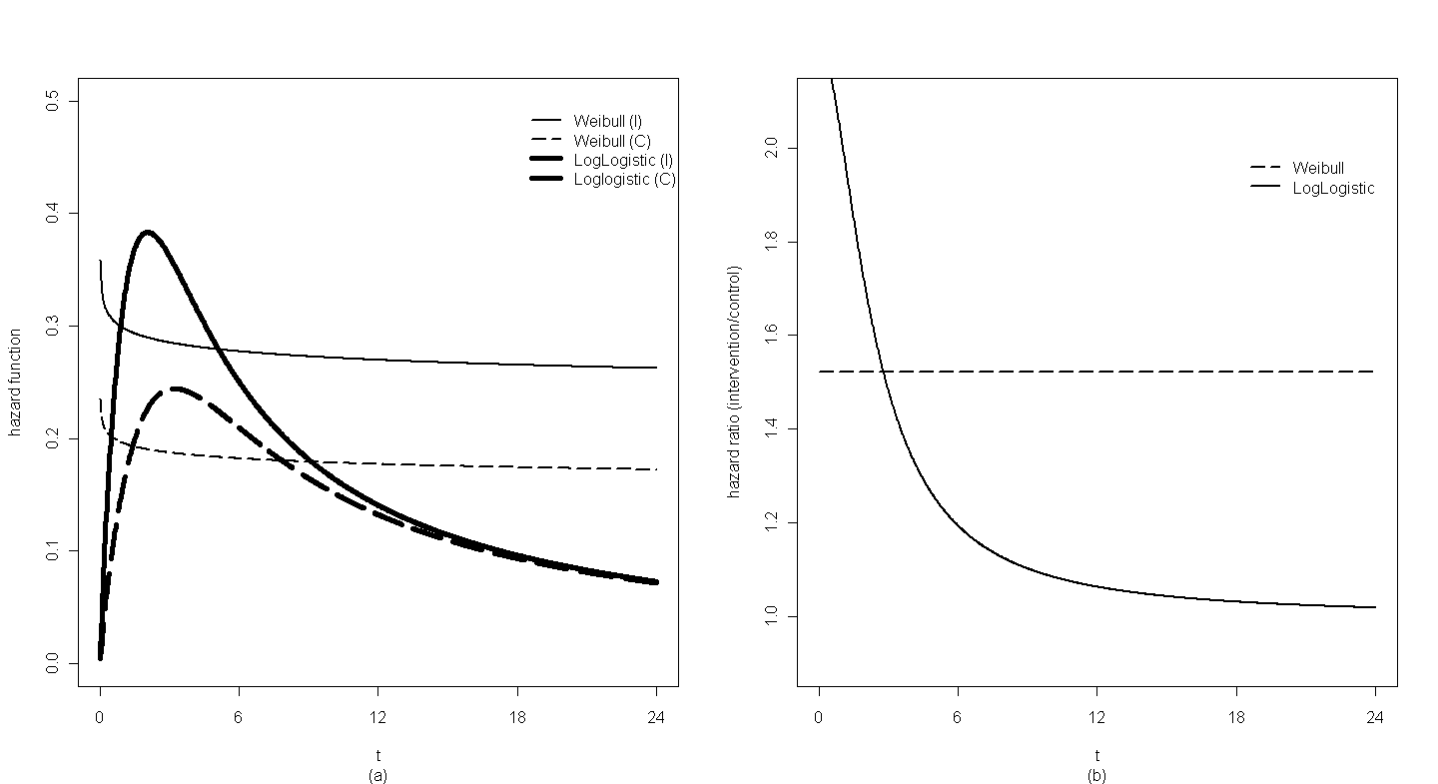
**Plot of Weibull and Log-logisitc hazard functions**. (a): Conditional hazard functions of the Weibull model and Log-logisitic model based on estimated parameter values for the intervention arm (I) and control arm (C). The Weibull hazard function is a monotonically decreasing function, whereas the log-logistic hazard funtion is a unimodal function that may better describe the natural history of HPV. (b): Conditional hazard ratio between the intervention and control arms from the two models. The Weibull model imposes a constant ratio, whereas the log-logistic model allows the clearance rate ratio to change over time.

Direct estimation of the coefficient parameters from model 1 by maximizing the likelihood may not be available with standard software such as SAS. To reduce the computation complexity, we further assume a parametric form on the clearance time for participants in the control arm, for example, conditional on the random effect * ξ*for a person in the control arm, let time to clearance of any HR-HPV infection have a Weibull distribution. That is *h*_0_(*t*) = *γα*_0_*t*^*γ*-1^, where *γ*(*γ *> 0) and *α*_0 _are the shape and scale parameters respectively for the Weibull distribution. The hazard function for Weibull distribution is a monotone function of *t *[13] (Figure 1a). The Gompertz distribution introduced for describing human mortality [4] is another parameteric distribution that has the proportional hazards property, and can be considered in the proportional hazards frailty model 1 .

Plugging *h*_0_(*t*) into expression 1, for the *j*th HR-HPV infection in person *i *with explanatory variables *x*_*ij*_, *j *= 1, 2, ⋯, *n*_*i*_, *i *= 1, 2, ⋯, *n*, the Weibull frailty model for clearance is:(2)

where the random frailty effect is assumed to follow a normal distribution with zero mean (i.e. exp (*ξ*_*i*_) ~log-normal distribution). Model 2 implies that conditional on the random frailty *ξ*_*i*_, the clearance of any HR-HPV infection follows a Weibull distribution with shape parameter *γ *and scale parameter , where circumcision's effect on clearance is quantified by its' coefficient parameter *β*. *ξ*_*i *_is the random effect shared by the multiple infections within the same person. Given a value of *ξ*_*i*_, the hazard ratio of HR-HPV clearance between the intervention and control arm is exp (*β*). Therefore, if *β *estimated from the data is significantly larger than 0, then exp(*β*) > 1, indicating the instantaneous clearance rate is larger if a participant was circumcised than not circumcised.

### Parametric Accelerated Failure Time Frailty Model

The Weibull frailty model given in 2 can be equivalently expressed in terms of the survivor function of the Weibull distribution as:(3)(4)

where *S*_0_(*t*) is the baseline survivor function of the conditional Weibull distribution, that is, *S*_0_(*t*) = exp(-*α*_0_*t*^*γ*^), where *α*_0 _= exp(*β*_0_). Thus we have .

Therefore, for a specific participant, the clearance process for an uncircumcised man is e^*β *^times of the clearance process if the man was circumcised, implying circumcision accelerates HR-HPV clearance with a factor of exp(*β*).

Conditional on the random effect, expression 4 corresponding to Weibull distribution in fact belongs to the family of parametric accelerated failure time (AFT) models [4]. For clustered survival data, the *general *AFT model with normal random frailty effect can be written as:(5)

where *ξ*_*i *_~Normal (0, *σ*^2^), *S*_0_(·) is the baseline survivor function of a parametric survival distribution, such as Weibull distribution, log-normal distribution, generalized gamma distribution, log-logistic distribution, generalized *F *distribution [13], and inverse Gaussian distribution [4]. Compared to parametric proportional hazards models where only few distributions have the proportional hazard property, this family of models comprehends a broader class of parametric survival distributions and allows for more flexibility on the shape of the conditional hazard function. It can be shown that AFT models can be expressed in log-linear model form [4], where it is easy to see that the predictor variables act additively on the logarithm of the survival time *T*, or equivalently multiplicatively on *T *itself. Conditional on a given value of the frailty effect *ξ*_*i *_in the AFT model 5, exp (*β*) has the interpretation of the ratio of the median (or any percentile) survival times between intervention and control arms. Moreover, Additional file 1 shows that the AFT frailty model also provides a marginal interpretation of the treatment effect, where -*β *is the average log ratio of the clearance times between intervention and control arms, i.e. .

It is important to notice that, however, except for the Weibull distribution (including the exponential distribution), other aforementioned distributions for AFT frailty models do not have the proportional hazards property and thus cannot be modeled as a proportional hazards frailty model given in 1, exp (*β*) consequently does not have the interpretation of conditional hazard ratio.

For the HR-HPV data, the proportional hazard frailty model 2 with Weibull distribution assumption implicitly imposes a constant instant clearance rate ratio (conditional on the random effect) over time between intervention arm and control arm (Figure 1b). The clearance rate ratio may however change over time. From Table 1, the majority of infections had cleared by year 2 in either arm, thus the clearance rate ratio should be close to 1; whereas the rate ratio by month 6 may be different from 1. The log-logistic distribution was also fit for the survivor function *S*(*t*|*ξ*_*i*_) in the AFT frailty model 5. The hazard of log-logistic distribution is a unimodal function of *t *when its shape parameter is larger than 1 (Figure 1a), which may better capture the natural history of HPV infection. It also allows the clearance rate ratio between arms to change over time(Figure 1b). The log-logistic AFT random frailty model is:(6)

where *a*_*ij *_= exp(*β*_0 _+ *x*_*ij *_β + *ξ*_*i*_), and *ξ*_*i *_~Normal (0, *σ*^2^).

### Estimation of model parameters

With parametric assumptions in model 1 or model 5, parameters (including fixed effects *β *and the variance of the random effect *σ*^2^) can be estimated using the maximum likelihood method. Recall that for the *j*th infection in the *i*th participant, the clearance time is observed to be in the interval of (*a*_*ij*_, *b*_*ij*_], where *a*_*ij *_= 0 for left censored observations and *b*_*ij *_= ∞ for right censored observations. For the *n*_*i *_genotype HR-HPV infections in person *i*, their clearances can either be left, right, or interval censored. Without loss of generality, let *l*_*i *_denote the number of left censored observations and *r*_*i *_denote the number of right censored observations, and hence *n*_*i *_-*l*_*i *_-*r*_*i *_is the number of interval censored observations on person *i*. Thus the full likelihood on all participants can be written as:(7)

where *S*_*ij*_(*t*|*ξ*_*i*_) is the conditional survivor function for the *j*th observation in person *i*, and given *ξ*_*i*_, the conditional survivor functions corresponding to the multiple infections within person *i *are independent.  is the density function for the normal random effect *ξ*_*i*_. The full likelihood is then obtained by integrating over all the possible values of the random effect.

The maximum likelihood estimate (MLE), denoted as  and , can be attained by maximizing the likelihood in expression 7 using iterative procedures, and the variance-covariance matrix is estimated as the inverse Hessian matrix. To test the significance of the explanatory variable, i.e. *H*_0 _: *β*_*k *_= 0, the likelihood ratio (LR) statistic (difference in -2Log(L) between the hierarchical models containing and not containing the variable) can be used by comparing it to a *χ*^2 ^distribution with 1 degree of freedom [14]. Alternatively, Wald type inference can be drawn with its MLE and variance. For the random effect parameter *σ*^2^, the null hypothesis *H*_0_: *σ*^2 ^= 0 corresponds to the situation where all of the observations within a cluster are independent, and thus may be of interest of testing. However, since *σ*^2 ^≥ 0,  = 0 is the boundary of the parameter space of *σ*^2^. [15] showed that the asymptotic distribution of the LR statistic (comparing between the models containing and not containing the random effect) is a 50:50 mixture of the  and  distribution. Thus a simple rule of computing P-value for testing *H*_0_: *σ*^2 ^= 0 is that if the LR statistic is 0, then *P *= 1; otherwise, the P-value is half of the P-value obtained from comparing the LR statistic to  distribution [16].

Since we assume a normal prior on the random frailty effect, the likelihood function normally does not have a close analytical form, although SAS PROC NLMIXED can numerically compute the integrals and maximize the approximated likelihood. The procedure provides commonly estimated statistics such as the MLE, Wald-type confidence intervals, and -2log-likelihood. In order to perform likelihood ratio inference for a variable, the hierarchical models with and without the variable have to be estimated respectively. The SAS code for analyzing the HR-HPV clearance data using model 2 is listed in Additional file 2 as an illustration, and relevant computation details are also discussed. The format of the input dataset and some useful options needed when calling the procedure are also described.

## Results of Study on Male Circumcision Effect on HR-HPV Clearance

Table 1 (upper panel) shows the enrollment prevalent infection profile for the intervention and the control arms. The number of HR-HPV infections (genotypes) per participant ranges from 1 to 5, indicating cluster size *n*_*i *_∈ [1,5]. The proportion of prevalent infections cleared by each follow-up visit is summarized in the bottom panel of Table 1. By the 6-month visit, 62.2% had cleared in the intervention arm and 58.0% had cleared in the control arm. At the end of the trial (24-month), the majority of the infections had cleared in both arms. It is of interest to see whether clearance was faster in the circumcised men. To estimate the effect of circumcision on HR-HPV clearance, the Weibull frailty model 4 and log-logistic AFT frailty model 6 were applied respectively, and the model parameters were estimated by maximizing the full likelihood given in equation 7. Table 2 lists the parameter estimates from the Weibull frailty model (left panel) and Log-logistic frailty model (right panel) obtained by PROC NLMIXED. The variance estimate for the random effect *ξ*_*i *_from the Weibull model is  = 0.88, and the likelihood ratio test on *H*_0_: *σ*^2 ^= 0 yields P-value < 0.0001. The corresponding estimate from the Log-logistic model is  = 0.85 (*P *= 0.002). Therefore, the clearance of multiple infections on the same individual is significantly correlated. Not accounting for the correlation will underestimate the standard error of the treatment effect estimate. The primary interest (circumcision's effect) is reflected by *β*_1_, and the two models yield very similar results. Although the shape of the conditional hazard functions from the two models is different (monotonically decreasing for the Weibull distribution and unimodal for the log-logistic distribution, Figure 1, the parameter estimates of interest from the two different models are highly comparable. From either model,  > 0 and the one-sided P-value = 0.02. Therefore, for each man, circumcision could significantly facilitate HR-HPV clearance should the man undergo circumcision. The median clearance time ratio is about 1.6 (95% CI, 0.9-2.2), implying clearance would be about 60% faster if a man was circumcised. HPV infection starts in epidermal basal cells and the virus then moves to epithelial surface [17], thus removal of foreskin physically removes a reservoire for viral replication, which may be one reason for the faster clearance.

**Table 2:** Parameter estimates from the Weibull and Log-logistic frailty model with normal random effect for studying circumcision's effect on HR-HPV clearance.

	Weibull				Log-logistic			
	**Est.**	**Std. Err.**	**95% C.I**.	**LR P-value (2-sided)**	**Est.**	**Std. Err.**	**95% C.I.**	**LR P-value (2-sided)**

	0.96	0.16	(0.63, 1.28)		1.78	0.28	(1.23, 2.33)	
	-1.59	0.34	(-2.25, -0.93)	< 0.0001	-1.30	0.17	(-1.63, -0.96)	< 0.0001
	0.42	0.23	(-0.02, 0.87)	0.04	0.45	0.22	(0.02, 0.89)	0.03
MSR^2^	1.56	0.35	(0.87, 2.24)		1.57	0.34	(0.90, 2.24)	

^The P-values were all obtained using likelihood ratio (LR) test.^

^1^γ is a nuisance parameter, thus no P-value is presented as no hypothesis testing was performed. ^2^MSR: median survival time ratio (conditional on the random effect).

^0^Weibull model: MSR = exp(*β*_1_); Log-logistic model: MSR = exp(*β*_1_).

There are several limitations of this application, however. One is that it was found that at the follow-up visits, a significant higher proportion of samples collected on circumcised men could not be amplified rendering more missing HPV results in intervention arm. In this illustration, it was assumed that the infection persisted during the interval if the testing result was missing for the follow-up visit at the end of the interval. This may underestimate circumcision's effect. Techniques for dealing with missing data, such as multiple imputation [18,19], can be utilized for this specific dataset. Another limitation for this study is that the follow-up intervals of 6, 12 and 24 months were too long to capture the clearance of HPV infection. It has been known that the median duration of genital HPV infection in woman is 8 months, and persistent detectable infection rate is approximately 30% after 1 year and 9% after 2 years [17]. In a recent observational study on men [20], it is reported that the median time to clearance was 5.9 months (95% CI, 5.7-6.1 months), and 75% infections had cleared by 24 months after initial detection, implying the clearance rate is low when *t *is large. With the effective immune response to HPV, most infections had cleared by 6 months as observed in the trial participants, meaning that there are no data describing the early phase of the clearance process. The limited data determine that there is "little to choose between alternative distributional models" [4] and different models may yield similar results in the range where data are observed. It is suggested to adopt the model "most convenient for the purpose in hand " [4]. However, any extrapolation on the functional form of the clearance process from the estimated model should be conducted with caution. This trial was primarily designed to study male circumcision's effect on preventing HIV acquisition. If it is of interest to examine HPV clearance in a future study, the study design should allow for a more frequent testing interval in order to capture the whole process. The presented parametric frailty models implicitly assume the same conditional baseline hazard functions for the clearance of different genotype infections within an individual. The different genotypes all belong to the papillomavirus family, and the mechanism of immune response is the same when fighting against the different type of HPV infections. Therefore it is reasonable to apply the same form of conditional baseline hazard functions for the clearance of different genotype infections.

## Conclusion and Discussion

Arbitrarily censored survival data is not uncommon in epidemiological studies, and the censoring nature should be considered during analysis to reduce estimation bias, or when the disease onset and diagnosis are two steps that need to be differentiated [7]. Moreover, clustered data may arise and the correlation within each cluster should also be accounted for. In the current study we particularly describe the use of parametric frailty models to explore treatment effect's on survival when data are clustered and subject to arbitrary censoring. The two main classes of models are parametric proportional hazards frailty model and accelerated failure time frailty model. Most commonly used survival distributions can be used in these models, providing abundant choices of parametric forms to appropriately model the data of interest. For example, Weibull distribution and Gamma distribution can be used for survival problems where the hazard monotonically changes with time, and log-logistic and log-normal distribution can be used when the hazard is a unimodal function of time. The main advantage of adopting a parametric form is for computational ease. On the other hand, with the presence of arbitrary censoring and clustering, it is difficult to perform model diagnostics on the assumption of the parametric form. A clear understanding of the scientific nature of the problem to be addressed is essential for choosing an appropriate parametric distribution in analysis. When using normal random effect, the presented models can be estimated using SAS PROC NLIMIXED, and the code for analyzing the example HPV dataset is provided in the Additional file 2. Models with random effects following other distributions may be estimated using PROC NLIMIXED by transforming the normal random effect using appropriate probability transformation function provided by SAS [21,22]. Alternatively, for gamma frailty model or log-t proportional hazards frailty model for data with arbitrary censoring, the "frailty()" function provided in the R package "survival" can be used.

Genital HPV infection has high prevalence in both men and women, and the high risk types of HPV are well known to be associated with anogenital cancers, especially cervical cancer [17]. Current diagnostic tools allow for simultaneous detection of multiple HPV genotypes, though the actual infection or clearance time is unknown. Therefore clustered data with arbitrary censoring are normally generated from such studies. The presented modeling approach can be used to study factors associated with HPV clearance (or persistence), or to compare the clearance process between different genotypes to examine type-specific persistence. However, as pointed earlier, the design for such studies need to allow for appropriate short testing intervals to capture the entire process.

## Competing interests

The authors declare that they have no competing interests.

## Authors' contributions

All authors made significant contributions to the proposed work, and have read and approved the manuscript. XK contributed to developing the study, analyzing and interpretation of the example dataset, as well as writing the manuscript. KJA, LHM and MCW contributed to the statistical analysis and interpretation, and RHG contributed to the interpretation and writing the manuscript.

## Pre-publication history

The pre-publication history for this paper can be accessed here:

http://www.biomedcentral.com/1471-2288/10/40/prepub

## Supplementary Material

Additional file 1 Log-linear form of the AFT frailty model Click here for file

Additional file 2 Preparation for estimation using SAS PROC NLMIXED, and the code for estimating male circumcision effect on HR-HPV clearance Click here for file
