# Supplementary material for: Parametric frailty models for clustered data with arbitrary censoring: application to effect of male circumcision on HPV clearance
Source: BMC Med Res Methodol. 2010 May 6;10:40. doi: 10.1186/1471-2288-10-40 (PMC2881064; doi:10.1186/1471-2288-10-40)
Supplement: Additional file 1 — Log-linear form of the AFT frailty model [file 1471-2288-10-40-S1.PDF]

Additional File 1: Log-linear form of the AFT frailty model

The AFT frailty model given in 5 can be equivalently expressed in the log-linear model form:

$$\log(T_{ij}) = \beta_0^* + \mathbf{x}_{ij}\beta^* + \delta\epsilon_{ij} + \xi_i \quad , \tag{8}$$

where $\xi_i \sim N(0, \sigma^2)$ is still the random frailty effect and is independent of ϵ_{ij}. ϵ_{ij}, as in a linear regression model, is the random error that models the random deviation of $\log(T_{ij})$ from the linear predictor part (conditional on ξ_i), and δ is a scale parameter for ϵ_{ij}. ϵ_{ij} is assumed to follow a certain known probability distribution [4], and different distributions of ϵ_{ij} correspond to different parametric forms for the survival time. For the Weibull frailty model in 4, the random error ϵ follows the Gumbel distribution [4], which has density function $f(\epsilon) = \exp(\epsilon - e^{\epsilon})$. For the Log-logistic frailty model in 6, the random error ϵ follows the logistic distribution with density function $f(\epsilon) = \frac{e^{\epsilon}}{(1+e^{\epsilon})^2}$. Using the parameterization in 5, parameters in model 8 can be expressed as $\delta = \frac{1}{\gamma}$ and $\beta^* = -\beta$.

With expression 8,recall that $x = 1$ denotes intervention and $x = 0$ denote control, thus we have:

$$\begin{aligned}
E[\log \frac{T(x=1)}{T(x=0)}] &= E[\log T(x=1) - \log T(x=0)] \\
&= E\left[E_{\xi}[\log T(x=1|\xi)]\right] - E\left[E_{\xi}[\log T(x=0|\xi)]\right] \\
&\text{plug in model 8} \\
&= E_{\xi}[\beta_0^* + \beta + \sigma\epsilon + \xi] - E_{\xi}[\beta_0^* + 0 + \delta\epsilon + \xi] \\
&= \beta^*
\end{aligned}$$

Therefore, model 8 has a population-level interpretation in that β^* is population average log ratio of clearance times between intervention arm and control arm.
