# Supplementary material for: Parametric frailty models for clustered data with arbitrary censoring: application to effect of male circumcision on HPV clearance
Source: BMC Med Res Methodol. 2010 May 6;10:40. doi: 10.1186/1471-2288-10-40 (PMC2881064; doi:10.1186/1471-2288-10-40)
Supplement: Additional file 2 — Preparation for estimation using SAS PROC NLMIXED, and the code for estimating male circumcision effect on HR-HPV clearance [file 1471-2288-10-40-S2.PDF]

Additional File 2: SAS PROC NLMIXED code for estimating male circumcision effect on HR-HPV clearance

To avoid non-convergence of the computation, it is helpful to first build a model without random effect where convergence normally is not of concern, and use the generated parameter estimates as initial values for the target model with random effect. Adaptive Gaussian quadrature (AGQ) [23] is the default method for approximating the integrals in PROC NLMIXED, and it is known to yield unbiased or least biased estimates on the fixed effect β and the variance of the random effect compared to methods based on pseudo-likelihood or Laplace approximation to the integral [23, 24]. A larger value of the number of quadrature points used in AGQ corresponds to a more precise approximation of the integral. The number of quadrature points Q used during execution of the procedure is reported in the output "Dimensions" Table. Due to approximation precision, different Q used may render different estimates on the parameters and likelihood [25]. It is advised to use at least $Q = 10$ quadrature points. Although the number of quadrature points for AGQ cannot be directly controlled in the procedure, one can adjust the tolerance level used in determining Q and the maximum value of Q allowed with options "QTOL" and "QMAX", respectively, in the procedure statement. The cost is a longer execution time, but was well tolerable for the HR-HPV example. Moreover, it was reported that in logistic regression model with random effect for binary data, different optimization procedures may also influence parameter estimates [25]. Therefore, it may be rational to explore the use of different optimization procedures with the "TECH" option, and make sure that the obtained parameter estimates from either procedure are global optimum of the likelihood function.

The input dataset should have one row for each HR-HPV genotype infection, thus a person with multiple infections have multiple rows. For the censored time to clearance, there should be two columns with one column recording the lower limit (start) of the interval during which clearance occurs and the other column recording the upper limit (end) of the interval. For left censored rows, only the upper limit values are observed and contribute to the likelihood given in 7. The values in the column of lower limit are not observed, however, they should not be coded as missing in the input dataset as they will be excluded by PROC NLMIXED before the procedure attempts to perform computation. Similarly, for the right censored rows, only the lower limit values contribute to the likelihood, yet the values in the column of upper limit should be assigned some non-missing values to assure execution of the estimation process. Another necessary column is the column that identifies the clusters, such as the participant identifier from the male circumcision trial in the HR-HPV example.

One limitation of SAS PROC NLIMIXED is that it only accommodates models with normal random effect. Models with random effects following other distributions, such as Gamma frailty model, can be estimated in PROC NLIMIXED by transforming the normal random effect using appropriate probability transformation functions provided by SAS [21]. Alternatively, for gamma frailty model or log-t proportional hazards frailty model for data with arbitrary censoring, the “frailty()” function provided in the R package “survival” can be used. The R package “survBayes” is another computation tool for interval censored survival data which uses a semiparametric Bayesian proportional hazards model [26].

```
PROC NLMIXED DATA=data_surv QMAX=100 QTOL=1E-6;
             PARMS gamma 0.55 b0 1.42 b1 -0.74;
             *Assigns parameters in the model optionally with initial values.
              The estimates from the model without the random frailty effect
              provide good starting values;
             BOUNDS gamma > 0; *The shape parameter for Weibull distribution;
             linp  = b0 + b1*(MC_group) +z ; *The linear predictor;
             alpha = exp(linp);
             S_low   = exp(-(alpha*time_low)**gamma);
             *Conditional survivor function at the lower limit of the
              interval;
             S_up   = exp(-(alpha*time_up)**gamma);
             *Conditional survivor function at the upper limit of the
              interval;
             ll = (censor="l")*log(1-S_up)
                  +(censor="r")*log(S_low)
                  +(censor="i")*log(S_low-S_up);
             *The log-likelihood function of the arbitrarily censored data as
              shown in expression 4;
             MODEL time_low ~ GENERAL(ll);
             *Specify the conditional log-likelihood given the random effect;
             *Note: dependent variable used here is not useful since the
              likelihood function was fully specified. It does not matter to
              use time_up or time_low or others, as long as the syntax is
              valid.;
             RANDOM z ~ normal(0,exp(2*logsig)) subject=id out=EB;
             *The random effect is normally distributed;
             ESTIMATE 'exp(beta)' exp(b1); *Estimating exp(beta);
 run;
```
